# The Inhibition of Gastric Cancer Cells’ Progression by 23,24-Dihydrocucurbitacin E through Disruption of the Ras/Raf/ERK/MMP9 Signaling Pathway

**DOI:** 10.3390/molecules27092697

**Published:** 2022-04-22

**Authors:** Huiping Liu, Huijuan Wang, Aijun Dong, Xiaoshuang Huo, Huaxiang Wang, Junchi Wang, Jianyong Si

**Affiliations:** The Key Laboratory of Bioactive Substances and Resources Utilization of Chinese Herbal Medicine, Ministry of Education, Institute of Medicinal Plant Development, Chinese Academy of Medical Sciences & Peking Union Medical College, Beijing 100193, China; l18610817598@163.com (H.L.); whj200428@163.com (H.W.); daj0938@163.com (A.D.); 13552646119@163.com (X.H.); whx9905@163.com (H.W.)

**Keywords:** 23,24-Dihydrocucurbitacin E, gastric cancer, ERK2, migration, invasion

## Abstract

Gastric cancer is considered to be one of the most common causes of cancer death worldwide due to its high recurrence and metastasis rates. The molecule 23,24-Dihydrocucurbitacin E (DHCE) is a cucurbitacin-derived tetracyclic triterpenoid compound that has anti-tumor activity, but the exact mechanism remains to be elucidated. This research aimed to explore the effects of DHCE on human gastric cancer cells and the possible mechanisms. The results showed that DHCE suppressed proliferation, migration, and invasion of gastric cancer cells, as well as induced apoptosis and G2/M phase arrest. Mechanistically, the potential targets and pathways of DHCE were predicted using database screening and verified using a molecular docking study, fluorescence staining, and Western blot. The results indicated that DHCE obviously inhibited the kinase activity of ERK2 via targeting its ATP-binding domain, destroyed F-actin microfilament, and reduced the expression levels of Ras, p-c-Raf, ERK, p-ERK, and MMP9 proteins. Collectively, our study demonstrated that DHCE suppressed gastric cancer cells’ proliferation, migration, and invasion through targeting ERK2 and disrupting the Ras/Raf/ERK/MMP9 signaling pathway. These properties make DHCE a promising candidate drug for the further design and development of novel and effective Ras/Raf/ERK/MMP9 pathway inhibitors for treating gastric cancer.

## 1. Introduction

Gastric cancer (GC) is the second leading cause of cancer-related deaths worldwide and ranks the first among all gastrointestinal cancers in terms of incidence [1]. Complete surgical resection is still the only chance to cure gastric cancer, but there is a possibility of recurrence even after complete resection [2]. In addition, due to absence of specific symptoms, most patients are diagnosed at an advanced stage and difficult to cure [3]. The two major features of gastric cancer are unlimited proliferation and metastasis, so many patients die not from primary cancer, but from cancer metastasis [4,5]. At present, the main treatment for metastatic gastric cancer is still cytotoxic chemotherapy. Even if multi-drug chemotherapy is used, the median survival time of patients with metastatic gastric cancer is still less than 1 year [6]. With the deepening understanding of the molecular mechanism of gastric cancer, the development of targeted drugs that can reduce toxicity and improve the survival rate of patients may become an important way to improve the prognosis of patients [7].

The development of cancer is a complex process caused by a battery of molecular aberrations, which lead to a variety of changes in signaling pathways. A deeper understanding of these molecular lesions will help target tumor-promoting molecules more effectively and develop less toxic and more effective anticancer therapies [8]. Abnormal activation of mitogen-activated protein kinases (MAPK) signaling pathways leads to cell differentiation, loss of apoptotic function, malignant transformation of cells, abnormal proliferation, tumor generation, enhanced invasion, and tumor metastasis. The MAPK pathway is constituted by four different cascades, covering extracellular signal-related kinase (ERK1/2), Jun amino-terminal kinase (JNK1/2/3), p38/MAPK, and ERK5 [9]. Among them, the MAPK/ERK pathway has been reported to be relevant to cell proliferation, differentiation, migration, and apoptosis [10]. The MAPK/ERK pathway contains different cascades, of which the Ras/Raf/ERK cascade is one of the most dysregulated cascades in human cancer [11]. Matrix metalloproteinase 9 (MMP9) is known as an important biomarker in the MAPK/ERK pathway [12]. MMP9 can induce extracellular matrix degradation, thereby reducing the stability of cancer cells and making them more prone to invasion and metastasis [13]. Therefore, it is necessary to study the function of the Ras/Raf/ERK/MMP9 pathway in tumor metastasis and invasion.

In recent years, natural products have drawn more and more attention in the drug development field because of their synergistic and attenuated effects [14,15]. Cucurbitacins are highly oxidized tetracyclic triterpenoids mainly isolated from Cucurbitaceae plants, and are divided into 12 classes from A to T with over 200 derivatives. In the plant kingdom, cucurbitacin B and D are the most common and are abundant in many plants, followed by E, G, H, and I [16]. In China, cucurbitacin prepared as tablets are mainly used as adjuvant treatment of chronic hepatitis and primary liver cancer. Previous research has indicated that cucurbitacins have cytotoxic, anti-inflammatory, and antitumor properties [17]. For instance, cucurbitacin E inhibited the proliferation of gastric cancer cells, with IC_50_ ranging from 80 to 130 nM after treatment for 48 h [18]. Cucurbitacin B inhibits immune regulation function and the inflammatory response of macrophages [19]. Cucurbitacin D induces G2/M phase arrest and apoptosis in Capan-1 pancreatic cancer cells through the ROS/p38 pathway [20]. At present, only a few types of cucurbitacins have been studied in depth, and even less of their molecular targets have been identified [21]. Therefore, it is necessary to expand the study of the biological activity and molecular mechanisms of natural cucurbitacin and its derivatives in the future. The molecule 23,24-Dihydrocucurbitacin E (DHCE) is a cucurbitacin-derived compound, whose biological activities have been rarely attended. Previous studies only reported the cytotoxic activity of DHCE against different cells, with an IC_50_ ranging from 0.93 to 36.20 μM after treatment for 48 h [22,23,24]. However, the mechanism of DHCE has not been reported.

In this research, we attempted to investigate the antitumor activity of DHCE in human gastric cancer cells. We revealed for the first time that DHCE inhibited the proliferation, migration, and invasion, and induced apoptosis and G2/M phase arrest in MGC803 and SGC7901 cells. Database screening and experimental verification showed that DHCE could inhibit the kinase activity of ERK2, destroy the cytoskeleton, and block the signal transduction of the Ras/Raf/ERK/MMP9 pathway. These findings therefore suggest that DHCE would serve as a potential candidate for targeting ERK2 and suppressing Ras/Raf/ERK/MMP9 pathway activation for anticancer.

## 2. Results

### 2.1. DHCE Selectively Inhibited the Proliferation of Gastric Cancer Cells

**Figure 1:**
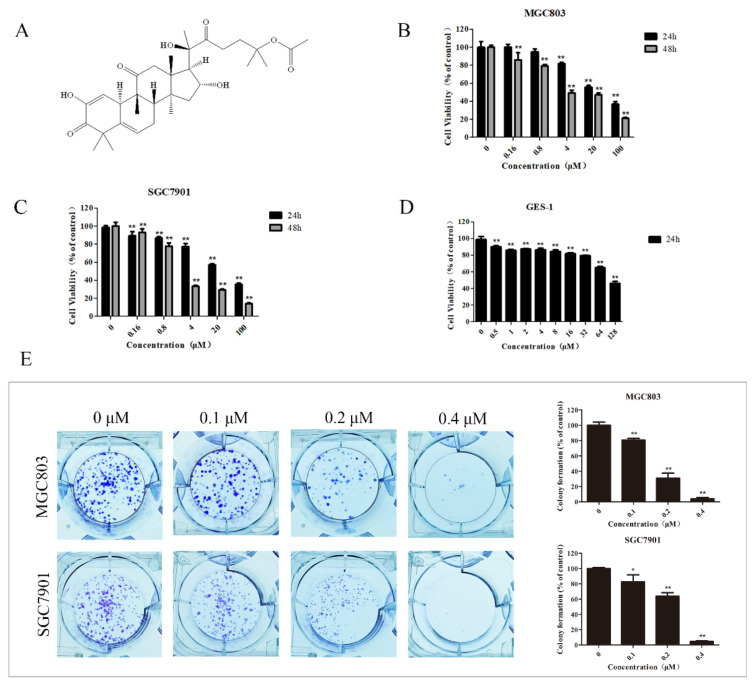
23,24-dihydrocucurbitacin E (DHCE) effectively suppressed the proliferation of gastric cancer cells. (**A**) Chemical structure of DHCE. (**B**,**C**) MTT assay of cell viability in MGC803 and SGC7901 cells treated with DHCE (0–100 μM) for 24 and 48 h, respectively. (**D**) MTT assay of cell viability in normal gastric epithelial (GES-1) cells treated with DHCE (0–128 μM) for 24 h. (**E**) Clones in MGC803 and SGC7901 cells treated with DHCE (0, 0.1, 0.2, or 0.4 μM) for 10 days in a colony formation assay and the statistic results of colony formation assays. The experiment was performed in triplicate, and the statistical results are expressed as means ± SD (* *p* < 0.05 and ** *p* < 0.01 compared with the control group).

The chemical structure of DHCE is exhibited in Figure 1A. To evaluate the anti-proliferation activity of DHCE on gastric cancer cells, MGC803 and SGC7901 cells were selected as the research objects. An MTT assay and clone formation assay were carried out to explore the effect of DHCE treatment on cell proliferation. As a result, the IC_50_ (half maximal inhibitory concentration) of DHCE on MGC803 cell were 36.98 ± 3.14 and 7.53 ± 1.07 μM after treatment for 24 and 48 h, respectively (Figure 1B). For SGC7901 cell, the IC_50_ values of DHCE were 34.01 ± 2.96 and 3.83 ± 0.49 μM after treatment for 24 and 48 h, respectively (Figure 1C). As the control, the IC_50_ value of DHCE on GES-1 cells, normal gastric epithelial cells, was more than 128 μM after treatment for 24 h (Figure 1D). These results showed that DHCE possessed significant cytotoxic activities toward MGC803 and SGC7901 cells but no obvious cytotoxicity in normal gastric epithelial cells. In addition, DHCE significantly reduced the clone formation ability of MGC803 and SGC7901 cells in a concentration-dependent manner (Figure 1E). These results together suggested that DHCE can inhibit the proliferation of GC cells.

### 2.2. DHCE Triggered Apoptosis in Gastric Cancer Cells

**Figure 2:**
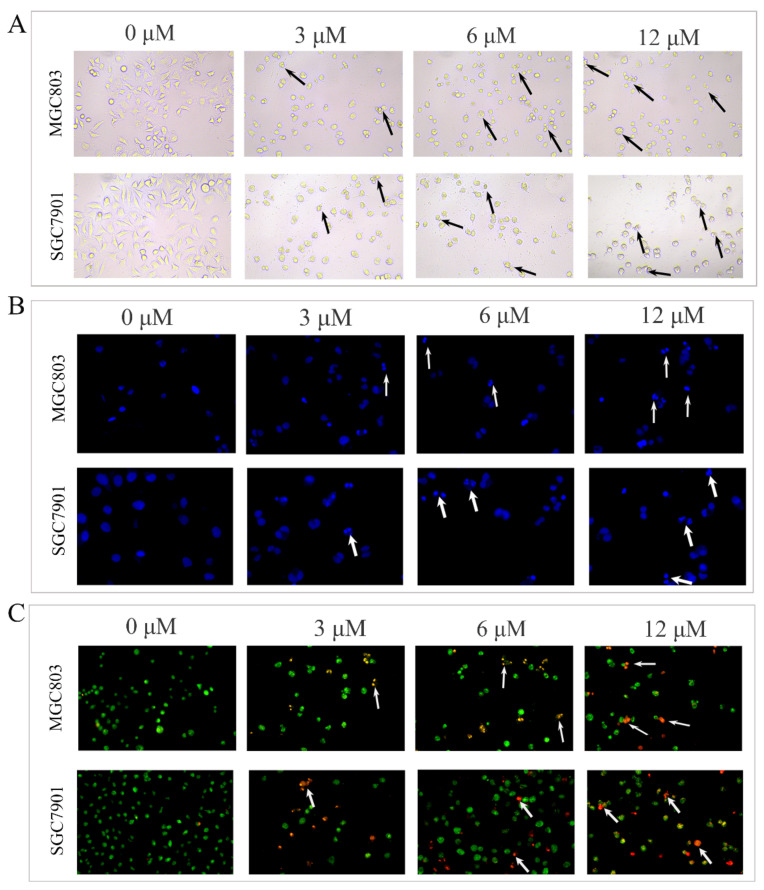
DHCE induced morphological changes of gastric cancer cells. (**A**) Morphology of MGC803 and SGC7901 cells treated with DHCE or not were observed under inverted microscope with a magnification of 200×. (**B**) Chromatin condensation and nuclear fragmentation were observed by DAPI staining under fluorescence inverted microscope with a magnification of 200×. (**C**) Normal cells, apoptotic cells, and necrotic cells were observed by AO/EB staining under a fluorescence inverted microscope with a magnification of 200×.

The results of cell proliferation experiments suggested that DHCE could inhibit the proliferation of MGC803 and SGC7901 cells. Due to the anti-proliferative effect being usually associated with apoptosis, the apoptosis-inducing effect of DHCE on the two cell lines was investigated. Firstly, the effect of DHCE on cell morphology of MGC803 and SGC7901 was examined under a fluorescent inverted microscope (Nikon, Japan). As exhibited in Figure 2A, the cells without DHCE treatment were flattened, well spread, and proliferated normally, while the cells with DHCE treatment displayed obvious cell detachment, cytoplasm condensation, cell shrinkage, and a disappearance of typical morphology. As shown in Figure 2B, through DAPI staining in the two cell lines, the chromatic agglutination and nuclear fragmentation characteristic of apoptotic cells were observed in the DHCE-treated cells. Meanwhile, cell apoptosis was also observed by AO/EB staining. As exhibited in Figure 2C, the nuclear chromatin of living cells was green with normal structure, and the nuclear chromatin of early apoptotic cells was yellowish green, condensed, or round. The nuclear chromatin of late apoptotic cells was orange-red, concentrated, or round. The chromatin of necrotic cells was orange-red with normal structure. AO/EB staining result indicated that the early apoptosis and late apoptosis gradually appeared in MGC803 and SGC7901 cells with the increase of DHCE concentration.

**Figure 3:**
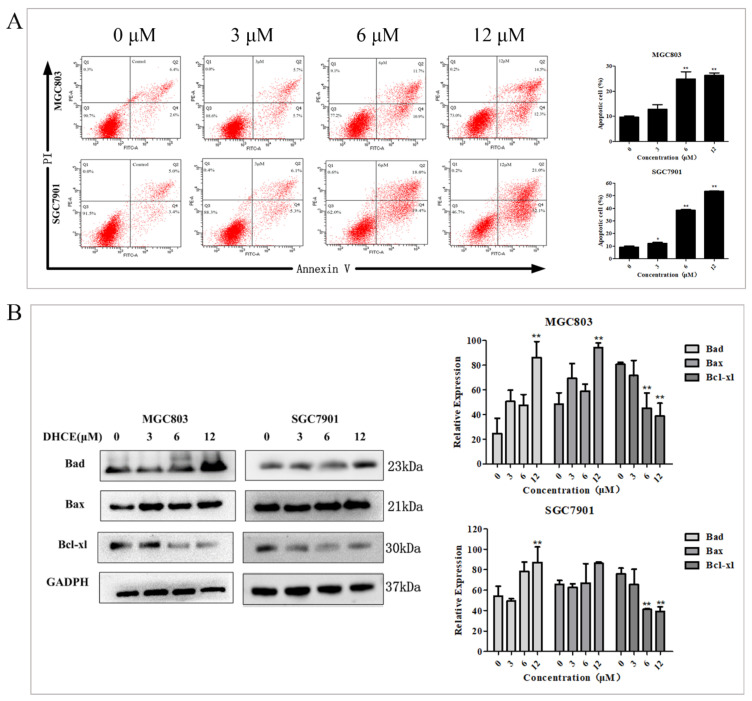
DHCE induced apoptosis of gastric cancer cells. (**A**) MGC803 and SGC7901 cells were treated with different concentrations of DHCE for 24 h, and the apoptosis level was detected by Annexin V-FITC/PI double staining. (**B**) Western blot was used to detect the expression levels of Bad, Bax and Bcl-xl in MGC803 and SGC7901 cells. The experiment was performed in triplicate, and the statistical results are expressed as means ± SD (* *p* < 0.05 and ** *p* < 0.01 compared with the control group).

Subsequently, double staining with Annexin V-FITC/PI was used to detect the apoptosis proportion of MGC803 and SGC7901 cells by flow cytometry. As exhibited in Figure 3A, the proportion of apoptotic cells increased with the increase of DHCE concentration. The expression levels of apoptosis-related proteins Bcl-xl, Bad and Bax in MGC803 and SGC7901 cells were detected by Western blot. The results indicated that DHCE treatment suppressed the expression of anti-apoptosis protein Bcl-xl and promoted the expression of pro-apoptosis protein Bad and Bax in the two cell lines (Figure 3B). These results suggested that DHCE induced apoptosis of GC cells.

### 2.3. DHCE Induces Gastric Cancer Cell G2/M Phase Arrest

**Figure 4:**
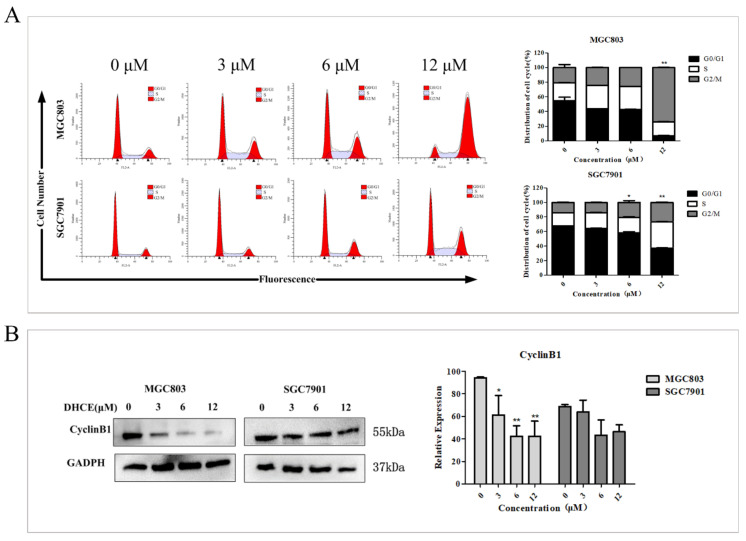
DHCE induced G2/M phase arrest of gastric cancer cells. (**A**) The distribution of MGC803 and SGC7901 cells at different phases after treatment with DHCE (0, 3, 6, or 12 μM) for 24 h. The experiment was performed in triplicate, and the statistical results are expressed as the means ± SD (* *p* < 0.05 and ** *p* < 0.01 compared with the control group). (**B**) The expression levels of cyclin B1 were detected by Western blot in MGC803 and SGC7901 cells which were treated with DHCE (0, 3, 6, or 12 μM) for 24 h. The experiment was performed in triplicate, and the statistical results are expressed as the means ± SD (* *p* < 0.05 and ** *p* < 0.01 compared with the control group).

Cell cycle disorder often leads to the uncontrolled proliferation of cancer cells, so blocking cell cycle helps prevent cancer cells from multiplying. To prove that DHCE inhibits the viability of MGC803 and SGC7901 cells through cell cycle arrest, the flow cytometric analyses were carried out and the results are shown in Figure 4A. In both MGC803 and SGC7901 cells, the number of cells blocked in G2/M phase increased in a DHCE concentration-dependent manner. Cell cycle progression is tightly regulated by cyclin-dependent kinases (CDKs) and cyclins. Cyclin B1 is significant in promoting G2/M transformation. Subsequently, the expression of cyclin B1 was detected in MGC803 and SGC7901 cells, and the results indicated that DHCE treatment reduced the expression level of cyclin B1 (Figure 4B).

### 2.4. DHCE Inhibits the Migration and Invasion of Gastric Cancer Cells

**Figure 5:**
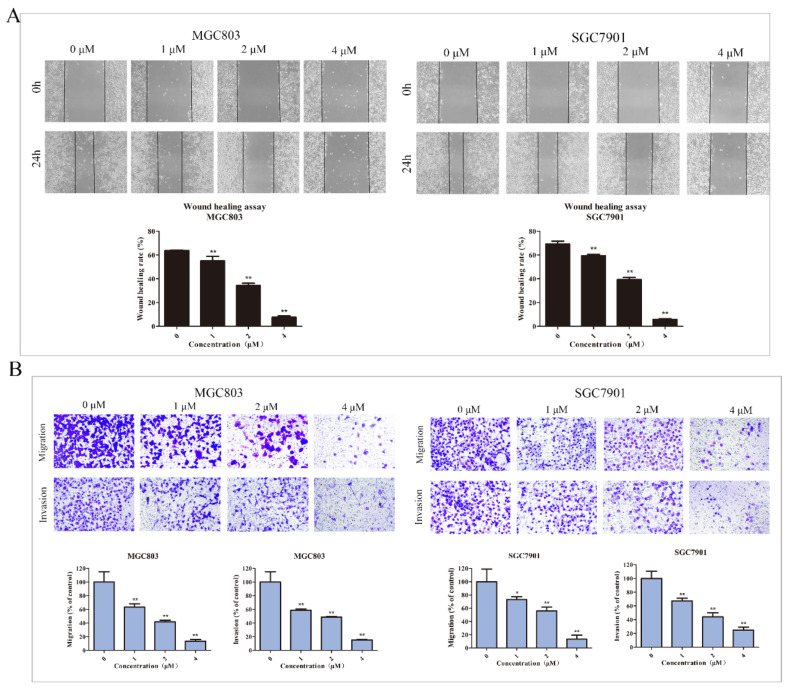
DHCE inhibited migration and invasion of gastric cancer cells. (**A**) The wound healing assay of MGC803 and SGC7901 cells. After making a linear wound, cells were treated with DHCE (0, 1, 2, or 4 μM) diluted with serum-free RPMI-1640 for 24 h. The photographs were captured at 0 and 24 h under an inverted microscope with a magnification of 100×. The wound area was measured, and the migration potential of each group was quantified. (**B**) Trans-well migration and invasion assay of MGC803 and SGC7901 cells. Images were photographed using an inverted microscope under a magnification of 200×. The number of invading cells were counted, and the migration and invasion potentials of each group were quantified. The experiment was carried out in triplicate, and the statistical results are expressed as the means ± SD (* *p* < 0.05 and ** *p* < 0.01 compared with the control group).

Cell migration and invasion are essential processes in cancer metastasis. Thus, inhibition of migration and invasion is an effective way for antitumor drugs to inhibit tumor progression. Therefore, the effect of DHCE on motility in MGC803 and SGC7901 cells was examined by a wound healing experiment. In order to remove the interference of cytotoxicity related effects, DHCE with low cytotoxicity concentrations was selected. As exhibited in Figure 5A, the wound healing rate in MGC803 cells was inversely correlated with DHCE concentration. At 24 h after scratching, the wound healing rate was 63.74% in the control group, and the value reduced to 34.41% in the 2 μM DHCE treatment group. Meanwhile, a consistent result was observed in SGC7901 cells, 24 h after scratch, the wound healing rate of the control group was 69.16%, and the value reduced to 39.29% in the 2 μM DHCE treatment group. It is noteworthy that the wound healing rates of both cell lines decreased to less than 10.00% after treated with 4μM DHCE for 24 h.

Subsequently, at the same concentration gradient, the trans-well chamber assay was carried out to further detect the inhibition effect of DHCE on the migration and invasion of these two cell lines. The results are shown in Figure 5B; in MGC803 cells, the number of cells invading to the inferior chamber in DHCE treatment groups were significantly less than that in the control group. At 4 μM, the migration and invasion rates were 13.60 and 15.38%, respectively. Meanwhile, the same effect was observed in SGC7901 cells. At 4 μM, the migration and invasion rates were 13.44 and 24.88%, respectively.

### 2.5. Network Pharmacology Analysis of DHCE Regulatory Signaling Networks

**Figure 6:**
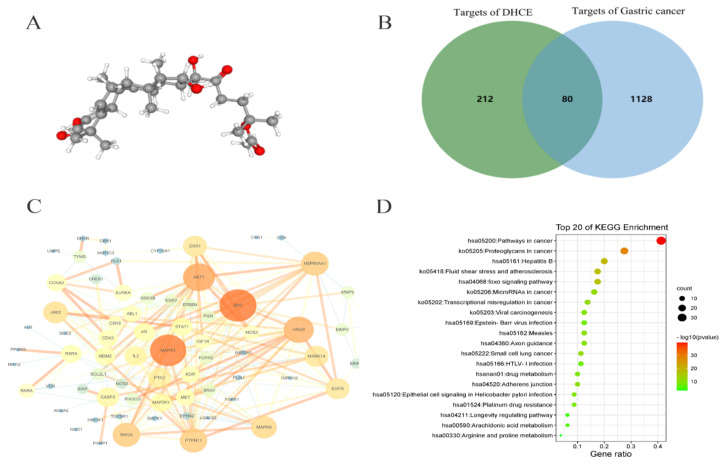
Targets and pathways enrichment analyses. (**A**) The 3D conformer of DHCE. (**B**) Venn diagram of 292 DHCE targets (left) and 1208 gastric cancer targets (right). Eighty common targets (middle) between DHCE and gastric cancer were identified as candidate targets for DHCE in the treatment of gastric cancer. (**C**) The PPI network of the 80 candidate targets. (**D**) KEGG pathway enrichment analysis of the 80 candidate targets. The size of dots indicates the number of genes in the KEGG pathways.

According to the 3D conformer of DHCE (Figure 6A), the PharmMapper database was used to screen the targets of DHCE, and a total of 292 potential targets of DHCE were searched. Meanwhile, the potential targets related to gastric cancer were retrieved from the OMIM, TTD, GeneCards, and DRUGBANK databases. After removing duplicate targets, 1208 potential targets relevant to gastric cancer was finally obtained. There were 80 common targets between 292 predictive targets of DHCE and 1208 gastric cancer-related targets (Figure 6B). Subsequently, 80 predicted targets were analyzed using the PPI network of STRING database, and their interaction and related functions were systemically realized. As exhibited in Figure 6C, in the PPI network, MAPK1 (a member of MAPK family, also known as ERK2) is the core predictive target, HRAS (a characteristic gene of RAS gene family) is located in the upstream of ERK2, and MMP9 is the downstream target of ERK2, which can degrade extracellular matrix.

In addition, to further disclose the underlying mechanism of DHCE, KEGG pathway enrichment analysis was conducted and the top 20 pathways were screened out by Metascape (Figure 6D). Among the pathways, “proteoglycans in cancer” and “adherens junction” were related to the migration and invasion of cancer cells. Previous studies had shown that changes in the expression of proteoglycans on tumor and stromal cell membranes affect the signal transduction, growth, survival, cell adhesion, migration, and angiogenesis of cancer cells [25]. In the KEGG pathway “proteoglycans in cancer”, the Ras/Raf/ERK/MMP9 cascade, mediated by proteoglycans, is involved in cell proliferation, survival, and angiogenesis. Besides, F-actin, a protein in the “adherens Junction” pathway, whose activation promotes cytoskeleton activation, and then promotes cell invasion and migration. Subsequently, the above targets and pathways predicted by network pharmacological were verified through experiments.

### 2.6. Molecular Docking between DHCE and ERK2

**Figure 7:**
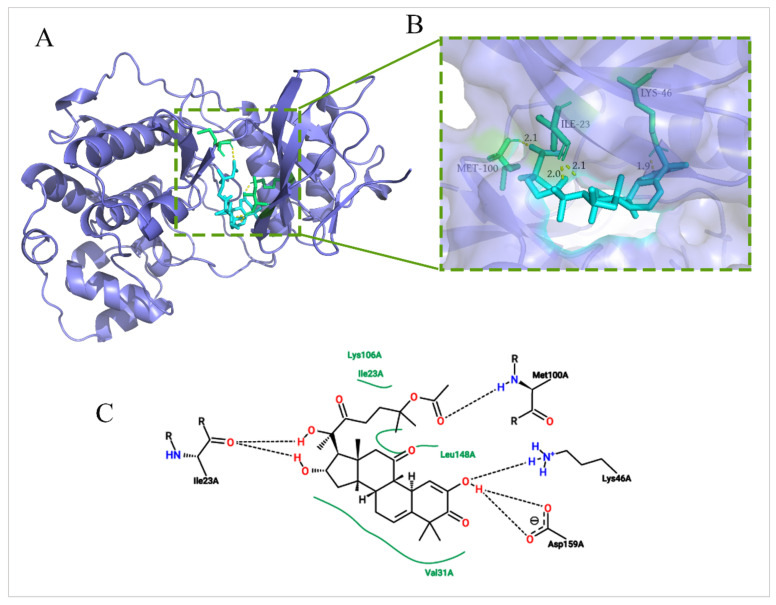
View of DHCE at the active site of ERK2. (**A**) Proposed binding mode of DHCE (in cyan) to the catalytic cleft of ERK2 (PDB ID: 5K4I). (**B**) Magnified view of DHCE interacting with residues around ERK2. (**C**) Two-dimensional diagrams for DHCE-ERK2 interactions generated from 3D models using the online tool Proteins Plus. Black dotted lines represent hydrogen bonds and the solid green lines represent hydrophobic interactions.

ERK2 was the core predictive target of the PPI network, and it was a key protein of the Ras/Raf/ERK/MMP9 pathway. Therefore, in order to verify the depression effect of DHCE on ERK2 protein, a molecular docking experiment was carried out. The molecular docking experiment was performed via AutoDockTools 1.5.6 and PyMol. The X-ray crystal structure of ERK2 with a resolution of 1.76 Å (Protein Data Bank, 5K4I) were used for docking studies. The greater the negative energy is, the larger the sum of the physical terms representing the combined free energy is. The lower the energy, the better the docking orientation. As shown in Figure 7A, B, DHCE bound to ERK2 at −8.3 Kcal/mol and fit nicely into the hydrophobic pocket to occupy the ATP binding site. Two-dimensional diagrams of DHCE interacting with surrounding residues of ERK2 were exhibited in Figure 7C. The results suggested that Lys106, Leu148, and Val31 had multiple hydrophobic interactions with DHCE, which helped stabilize their binding. DHCE formed a hydrogen bond with Met100 and Lys46, and it formed two hydrogen bonds with Ile23 and Asp159. The formation of hydrogen bonds improved the ability of ligand to target protein and thus better inhibited ERK2 protein. Ile23 is a crucial amino acid, which plays a particular role in stabilizing DHCE–ERK2 binding, supplying both hydrogen bonding and hydrophobic forces. The results of molecular docking suggested that the inhibition of DHCE on ERK2 depended on both hydrogen bonds and hydrophobic forces.

### 2.7. DHCE Destroys F-actin Microfilament and Attenuates Ras/Raf/ERK/MMP9 Pathways in Gastric Cancer Cells

**Figure 8:**
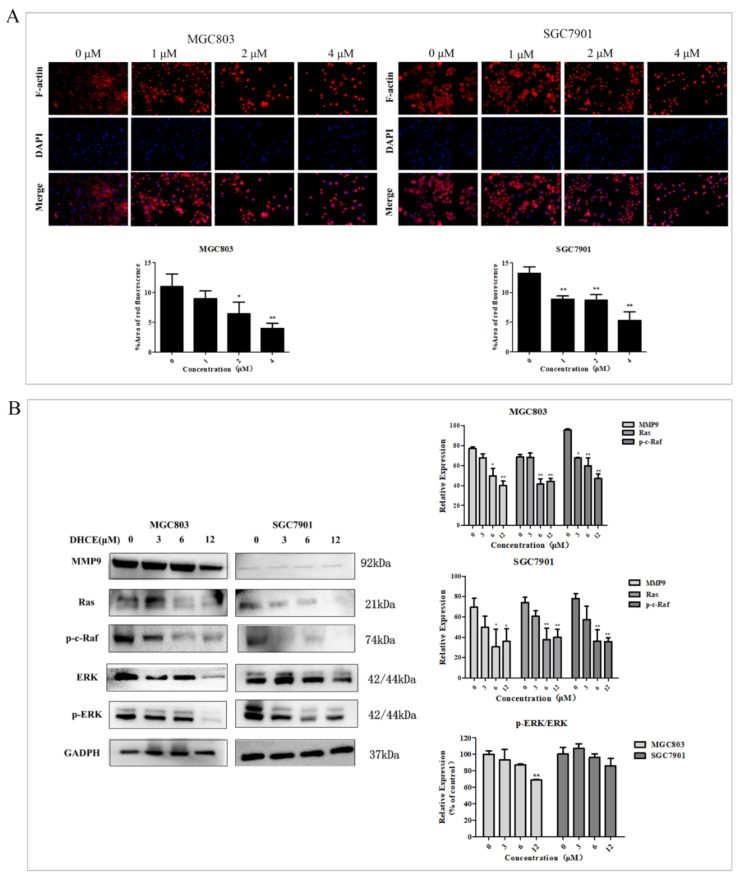
DHCE destroys cytoskeletal organization and attenuates the Ras/Raf/ERK/MMP9 pathway in gastric cancer cells. (**A**) The expression of F-actin microfilament was measured by Actin-Tracker Red-555 staining. The relative expression of F-actin microfilament was quantified by red fluorescence area. (**B**) Immunoblot analyses of MMP9, Ras, p-c-Raf, and p-ERK/ERK in MGC803 and SGC7901 cells treated with or without DHCE. The experiment was performed in triplicate, and the statistical results are expressed as the means ± SD (* *p* < 0.05 and ** *p* < 0.01 compared with the control group).

The cytoskeleton system consists of microfilament, microtubule, and intermediate filament, of which microfilament is related to cell movement and contraction. Actin microfilaments (F-actin) are a vital part of the cytoskeleton and crucial to the cell morphology and motility of eukaryotic cells [26]. In order to further verify that DHCE inhibits the migration and invasion of MGC803 and SGC7901 cells by destroying the cytoskeleton, the expression levels of F-actin microfilament in MGC803 and SGC7901 cells were detected by using Actin-Tracker Red-555 staining. The results showed that the microfilaments of DHCE-untreated groups were intact in both cell lines, while the microfilaments of DHCE-treated groups were broken and aggregated (Figure 8A). These results indicated that DHCE could suppress the migration and invasion of GC cells by destroying microfilaments.

Targets and pathways analysis showed that the Ras/Raf /ERK/MMP9 cascade is essential to the growth, proliferation, survival, invasion, and migration of gastric cancer cells. The expression of Ras, p-c-Raf, ERK, p-ERK, and MMP9 proteins in MGC803 and SGC7901 cells treated with or without DHCE were detected by Western blot. As exhibited in Figure 8B, DHCE treatment significantly inhibited the expression of these proteins when compared with the control group, as well as reduced the ratio of p-ERK to ERK. The results suggested that DHCE inhibits the migration and invasion of MGC803 and SGC7901 cells by inhibiting Ras/Raf/ERK/MMP9 pathway activation.

## 3. Discussion

DHCE is a natural product that can be isolated from the root of *Siraitia grosvenorii*. In this study, we attempted to identify the anti-cancer effect of DHCE and proposed that DHCE may be an inhibitor of gastric cancer cells. It has been reported that DHCE can inhibit the proliferation of gastric cancer BGC823 cells with an IC_50_ of 36.20 ± 0.94 μM for 48 h [23]. In the study, DHCE treatment for 48 h noticeably suppressed the proliferation of gastric cancer MGC803 and SGC7901 cells with IC_50_ values of 7.53 ± 1.07 and 3.83 ± 0.49 μM, respectively, indicating that DHCE has different sensitivity to different cell lines. Furthermore, Inhibition of proliferation is often associated with increased apoptosis and inhibition of invasion and migration [27]. Since DHCE can significantly inhibit the proliferation of MGC803 and SGC7901 cells, the effects and underlying mechanisms of DHCE on apoptosis, invasion, and migration of gastric cancer cells were further explored.

Previous studies have shown that cell cycle disorder leads to uncontrolled cell proliferation, which is a feature of cancer; blocking the cell cycle inhibits cell proliferation [28]. Additionally, the imbalance between pro-apoptotic proteins, such as Bad and Bax, and anti-apoptotic proteins, such as Bcl-2 and Bcl-xl can lead to mitochondrial membrane permeability damage, triggering the formation of apoptotic bodies, and ultimately leading to apoptosis [29]. Therefore, apoptosis induction and cycle arrest of cancer cells are effective strategies for cancer therapy [30,31]. In this study, DHCE-treated gastric cancer cells showed chromatin shrinkage and nuclear fragmentation, which are typical apoptotic morphologies [32]. With the increase of DHCE concentration, the proportion of apoptotic cells increased by up-regulating the expression of the pro-apoptotic proteins Bad and Bax, and down-regulating the expression of anti-apoptotic protein Bcl-xl. Besides, DHCE treatment decreased the expression level of cyclinB1 protein and induced G2/M cycle arrest in gastric cancer cells. Therefore, the increased apoptosis and G2/M phase arrest might be responsible for gastric cancer cell proliferation inhibition by DHCE.

Gastric cancer is prone to metastasis; some patients have lymph node metastasis or even distant organ metastasis at the initial diagnosis, which affects the prognosis and survival rate of patients [33,34]. The invasion and migration of cancer cells are influenced and regulated by many biomolecules and signaling pathways [35], so finding effective signaling pathway inhibitors can help inhibit tumor metastasis and improve therapeutic effects. So far, network pharmacology has been applied to targets prediction and pharmacological mechanisms for numerous bioactive molecules based on the interactions of “disease–gene–target–drug” [36,37]. In this study, the results of network pharmacology showed that KEGG pathway “proteoglycans in cancer” were related to the migration and invasion of cancer cells. Additionally, the Ras/Raf/ERK/MMP9 cascade in “proteoglycans in cancer” is involved in cell proliferation, survival, and angiogenesis. Besides, F-actin is a protein in the KEGG pathway “Adherens junction”, whose activation promotes cytoskeleton activation, and then promotes cell invasion and migration.

Research has shown that Ras is an important regulatory component involved in normal cell proliferation, differentiation, and malignant transformation. Oncogenic Ras activation affects a variety of cellular processes through its broad signaling pathways, including inhibiting apoptosis and promoting proliferation [38]. The MAPK/ERK pathway is crucial in cancer development as a downstream signaling cascades of Ras [39], which affects many cellular processes, including proliferation, apoptosis, migration, and invasion, and its activation promotes the carcinogenesis of human genes [40,41]. The overexpression of MMPs is related to the migration and invasion of cancer cells; the activation of ERK can lead to the overexpression of MMPs in human cancer which leads to tumor metastasis [42,43]. In this study, the migration and invasion of MGC803 and SGC7901 cells were significantly inhibited by low-concentration DHCE treatment. It was confirmed that this inhibition effect was due to DHCE destroying F-actin microfilament and down-regulating the protein expression of Ras, p-c-Raf, and MMP9, and reduced the ratio of p-ERK/ERK. In addition, molecular docking results showed that DHCE could bind to ERK2 stably with a binding energy of −8.3 Kcal/mol, suggesting that DHCE may act as an inhibitor of ERK2 and block downstream signal transmission of ERK2.

In short, DHCE destroyed F-actin microfilament to block cytoskeleton activation, and inhibited the protein expression of the Ras/Raf/ERK/MMP9 pathway to block the signal transmission of this pathway, thereby inhibiting the migration and invasion of gastric cancer cells.

## 4. Materials and Methods

### 4.1. Reagents and Antibodies

DHCE was isolated from dichloromethane extracts of the roots of *Siraitia grosvenorii,* and it was dissolved in dimethyl sulfoxide (DMSO) to make a 40 mM stock solution. The ^1^H-NMR (600 MHz, pyridine-*d*5) spectrum of DHCE is shown in Figure S1. The ^13^C-APT (150 MHz, pyridine-*d*5) spectrum of DHCE is shown in Figure S2.

RPMI 1640, DMEM, and 0.25% Trypsin-EDTA were purchased from Gibco (Grand Island, NY, USA). MTT, Penicillin–Streptomycin liquid, and AO/EB were purchased from Solarbio (Beijing, China). Crystal Violet Staining Solution, Annexin V-FITC Apoptosis Detection Kit, Actin-Tracker Red-555, DAPI, and BCA Protein Assay Kit were purchased from Beyotime (Shanghai, China). Matrigel matrix was purchased from Corning Inc. (Corning, NY, USA). Fetal bovine serum (FBS) was purchased from EVERY GREEN (Zhejiang, China). Antibodies against MMP9, Cyclin B1, and Bad were purchased from ABclonal Technology (Wuhan, China). Ras, p-c-Raf, ERK1/2, p-ERK1/2, and GADPH were purchased from Cell Signaling Technology (Danvers, MA, USA). Bcl-xl, Bax, and goat anti-rabbit HPR-conjugated secondary antibody was obtained from Boster Biological Technology (Wuhan, China).

### 4.2. Cell Lines and Cell Culture

The human gastric cancer cell lines MGC803, SGC7901, and human normal gastric epithelial cell GES-1 were all obtained from Basic Medicine Cell Center, Chinese Academy of Medical Sciences (Beijing, China). MGC803 and SGC7901 cells were maintained in RPMI-1640 with 10% FBS and 1% penicillin/streptomycin in a humidified atmosphere at 37 °C with 5% CO_2_. GES-1 cells were cultured in DMEM containing 10% FBS and 1% penicillin/streptomycin under the same conditions.

### 4.3. Cell Viability Assay

Cell viability was evaluated by MTT assay. Cells in the logarithmic phase were digested with trypsin, and 100 μL cell suspension (approximately 3 × 10^3^ cells/well) was added into 96-well plates. After overnight incubation, the cultured cells were treated with different concentrations of DHCE for 24 or 48 h. Thereafter, 10 μL MTT (5 mg/mL) was added to each well and incubated at 37 °C for 4 h. Then, the medium was removed and 150 μL of DMSO/well was added to dissolve the formazan crystals. The absorbance was measured at 570 nm using a microplate reader (Infinite M1000 Pro, Tecan, Switzerland).

### 4.4. Colony Formation Assay

To analyze the effects of DHCE on colony formation, single cells were inoculated in 6-well plates (400 cells/well) and cultured for 24 h to allow cells to adhere. Then the cells were routinely cultured for 10 days with various concentrations of DHCE (0, 0.1, 0.2, or 0.4 μM). After washing with PBS, the colonies were fixed with methanol, stained with crystal violet, and then counted.

### 4.5. Observation of Cell Morphological Changes

MGC803 and SGC7901 cells were plated into 24-well plates, 1 mL per well, at a density of 3 × 10^4^ cells/mL. After overnight incubation, DHCE of different concentrations (0, 3, 6, or 12 μM) were added and incubated for 24 h; an inverted microscope (Olympus, Tokyo, Japan) was used to observe the cellular morphology. After that, cells were washed with PBS and fixed with 4% paraformaldehyde solution for 20 min, then stained with DAPI for 30 min in the dark. As for AO/EB staining, the cells treated with DHCE under the same conditions were rinsed with PBS and stained in the dark for 5 min. The cellular morphology was observed under a fluorescent inverted microscope (Nikon, Japan).

### 4.6. Apoptosis Analysis by Flow Cytometry

MGC803 and SGC7901 cells were seeded into six-well culture plates. After incubation for 24 h, the cells were treated with DHCE (0, 3, 6, or 12 μM) for another 24 h. After being harvested and washed twice with PBS, the cells were stained with an Annexin V-FITC Apoptosis Detection Kit as directed. The stained cells were detected and analyzed by an FACS Calibur Flow cytometer (Becton Dickinson, CA, USA).

### 4.7. Cell Cycle Analysis by Flow Cytometry

The MGC803 and SGC7901 cells were inoculated into 6-well culture plates and treated with DHCE (0, 3, 6, or 12 μM) for 24 h. Then the cells were harvested and fixed with 70% cold ethanol overnight at 4 °C. The fixed cells were centrifuged to remove the ethanol and rinsed twice with PBS. The suspended cells were stained with 500 μL PI solution containing RNase A in the dark for 30 min. AnFACS Calibur Flow cytometer (Becton Dickinson, CA, USA) was used for samples’ detection and ModFit LT 4.0 software was used for data analysis.

### 4.8. Wound Healing Assay

The cells in logarithmic phase were plated into 24-well plates and cultured to approximately 90% confluent, and a linear wound was made in the monolayer cells with a sterile 200 μL pipette tip. After the unattached cells and debris being removed by rinsing with PBS, the cells were treated with different concentrations of DHCE (0, 1, 2, or 4 μM) for 24 h. Images of the wound were captured at 0 and 24 h under an inverted microscope (Olympus, Tokyo, Japan) with a magnification of 100×. The area of the wound was measured by Image J 1.53a software (National Institutes of Health, Bethesda, MD, USA). The wound healing rates were calculated according to the change of wound area.

### 4.9. Cell Migration and Invasion Assays

The cell invasion and migration assays were carried out using trans-well chambers coated with or without Matrigel. In brief, the cells (3 × 10^4^ per well) were inoculated in the upper chamber without Matrigel for migration experiments or precoated with 70 μL Matrigel for invasion experiments, and lower chamber wells were filled with 600 μL RPMI-1640 medium supplemented with 10% FBS and various concentrations of DHCE (0, 1, 2, or 4 μM). After 24 h incubation, invasive and migratory cells were fixed with methanol for 20 min, and stained using crystal violet for 30 min. Images were captured using an inverted microscope (Olympus, Tokyo, Japan) with a magnification of 200×. The migration or invasion rate was calculated according to the number of invaded cells.

### 4.10. Potential Target Prediction

The 3D conformer of the compound was downloaded from PubChem database (https://pubchem.ncbi.nlm.nih.gov) (Accessed on 21 April 2021) and uploaded to the PharmMapper database (http://www.lilab-ecust.cn/pharmmapper) (Accessed on 21 April 2021) to predict potential drug targets. The official names of the targets were taken from the UniProt database (http://www.uniprot.org) (Accessed on 2 May 2021) by confining the species to “Homo sapiens”. The known gastric cancer-related targets were retrieved from the GeneCards (https://www.genecards.org) (Accessed on 5 May 2021), OMIM (https://www.omim.org) (Accessed on 5 May 2021), TTD (http://db.idrblab.net/ttd) (Accessed on 5 May 2021), and DRUGBANK database (https://go.drugbank.com) (Accessed on 5 May 2021). The common targets between the potential targets of DHCE and gastric cancer-associated targets were selected as the candidate targets. The relationship of drug targets and gastric cancer targets was described in a Venn diagram. The protein–protein interaction (PPI) network was calculated on the web of the Retrieval of Interacting Genes (STRING) database (https://string-db.org) (Accessed on 8 May 2021) and Cytoscape (version 3.7.1) software was also utilized to visualize the molecular interaction networks. Metascape (https://metascape.org/gp/index.html) (Accessed on 10 May 2021) platform was hired for Kyoto Encyclopedia of Genes and Genomics (KEGG) pathway enrichment analysis. When *p*-value ≤ 0.01, the KEGG pathways were considered to be statistically significant. Then the top 20 KEGG pathways were chosen for further analysis.

### 4.11. Molecular Docking Calculations

The binding effect of DHCE to ERK2 was estimated by molecular docking. In short, molecular docking was performed between DHCE and ERK2 and the crystal structure of ERK2 was collected from Protein Data Bank (ID:5K4I) [44]. The missing residues (172–189) were constructed and refined using Chimera1.16 and Modeller10.2 software. The AutoDockTools 1.5.6 software was used to prepare ERK2 and DHCE before docking. After verification of the docking parameters, AutoDockTools 1.5.6 was used for docking calculations of ERK2 and DHCE, and Lamarckian genetic algorithm was used. The docking grid contains the ATP-active site of ERK2 receptor with a grid size of 70 Å × 70 Å × 70 Å and a spacing value of 0.375 Å. Other interconnection parameters were kept to the default values. The grid center was placed at 13.705, −0.511, 16.724 (XYZ coordinates). The binding interaction of DHCE with ERK2 was visualized by PyMol and Proteins Plus.

### 4.12. Cytoskeleton Staining and Observation

Cells were inoculated on glass coverslips in 48-well plates at a density of 1.5 × 10^4^ cells/well. After incubation overnight, cells were treated with DHCE (0, 1, 2, or 4 μM) for 24 h, and then fixed with 4% paraformaldehyde for 30 min. After rinsing with PBS twice, the cells were permeabilized with 0.1% Triton X-100 for 30 min at room temperature, then Actin-Tracker Red-555 staining solution was added for 1 h at room temperature. Then, the nucleus was counterstained by DAPI for 30 min and the cells were photographed under an inverted fluorescent microscope (Nikon, Japan).

### 4.13. Western Blot Analysis

MGC803 and SGC7901 cells were plated in Petri dishes with a diameter of 10 cm and exposed to DHCE for 24 h after overnight culture. Then, the cells were harvested and lysed. The protein concentrations of supernatant were measured by BCA assay kit. Then, equal amount of extracted protein (50 μg) from each sample was separated by SDS-PAGE gel and electrically transferred onto PVDF membranes. After being blocked with 5% skim milk solution for 2 h at room temperature, the membranes were incubated with the corresponding primary antibodies at 4 °C overnight. Subsequently, the primary antibody was washed thrice with TBST, then the HRP-conjugated secondary antibodies was incubated at room temperature for 1 h. Protein bands were visualized by ECL Western blotting analysis system (CWBIO, Beijing, China). Image Lab 3.0 software was used to quantify the intensity of the bands.

### 4.14. Statistical Analysis

All experiments were repeated three times independently, and results are represented as the means ± SD. The SPSS 22.0 (IBM Corp., New York, NY, USA) and GraphPad Prism 5.0 software (GraphPad Software, La Jolla, CA, USA) were used for statistical analysis. One-way analysis of variance (ANOVA) was used to detect the significance between groups. A *p*-value of less than 0.05 (*p* < 0.05) was considered to indicate a statistically significant difference.

## 5. Conclusions

In summary, we revealed that DHCE can suppress proliferation, induce apoptosis and G2/M phase arrest in gastric cancer cells, and suppress the migration and invasion of gastric cancer cells by targeting the Ras/Raf/ERK/MMP9 pathway. Our study proposed a potential mechanism by which DHCE inhibited the migration and invasion of gastric cancer cells. It will be a drug candidate for the design and development of gastric cancer therapeutic inhibitors targeting the Ras/Raf/ERK/MMP9 pathway.

## Data Availability

Not applicable.

## Supplementary Materials

The following are available online at https://www.mdpi.com/article/10.3390/molecules27092697/s1, Figure S1: ^1^H-NMR (600 MHz, pyridine-d5) spectrum of 23,24-dihydrocucurbitacin E, Figure S2: ^13^C-APT (150 MHz, pyridine-d5) spectrum of 23,24-dihydrocucurbitacin E, Figure S3: Original western blots images of MMP9, ERK, Bcl-xl and Bax in MGC803 cells (*n* = 3), Figure S4: Original western blots images of p-c-Raf and Cyclin B1 in MGC803 cells (*n* = 3), Figure S5: Original western blots images of p-ERK and Bad in MGC803 cells (*n* = 3), Figure S6: Original western blots images of Ras in MGC803 cells (*n* = 3), Figure S7: Original western blots images of ERK, Bcl-xl and Bax in SGC7901 cells (*n* = 3), Figure S8: Original western blots images of p-c-Raf and Ras in SGC7901 cells (*n* = 3), Figure S9: Original western blots images of p-ERK in SGC7901 cells (*n* = 3), Figure S10: Original western blots images of Bad in SGC7901 cells (*n* = 3), Figure S11: Original western blots images of Cyclin B1 in SGC7901 cells (*n* = 3), Figure S12: Original western blots images of MMP9 in SGC7901 cells (*n* = 3).
