# Supplementary material for: The Inhibition of Gastric Cancer Cells’ Progression by 23,24-Dihydrocucurbitacin E through Disruption of the Ras/Raf/ERK/MMP9 Signaling Pathway
Source: Molecules. 2022 Apr 22;27(9):2697. doi: 10.3390/molecules27092697 (PMC9100127; doi:10.3390/molecules27092697)
Supplement: Supplementary file 1 [file molecules-27-02697-s001.zip › molecules-1654701-supplementary.pdf]

The Inhibition of Gastric Cancer Cells' Progression by 23,24-Dihydrocucurbitacin E through Disruption of the Ras/Raf/ERK/MMP9 Signaling Pathway

Huiping Liu, Huijuan Wang, Aijun Dong, Xiaoshuang Huo, Huaxiang Wang, Junchi Wang*, Jianyong Si*

The Key Laboratory of Bioactive Substances and Resources Utilization of Chinese Herbal Medicine, Ministry of Education, Institute of Medicinal Plant Development, Chinese Academy of Medical Sciences & Peking Union Medical College, Beijing, 100193, China; l18610817598@163.com (H.L.); whj200428@163.com (H.W.); daj0938@163.com (A.D.); 13552646119@163.com (X.H.); whx9905@163.com (H.W.)

*Correspondence: jcwang@implad.ac.cn (J.W.); jysi@implad.ac.cn (J.S.)

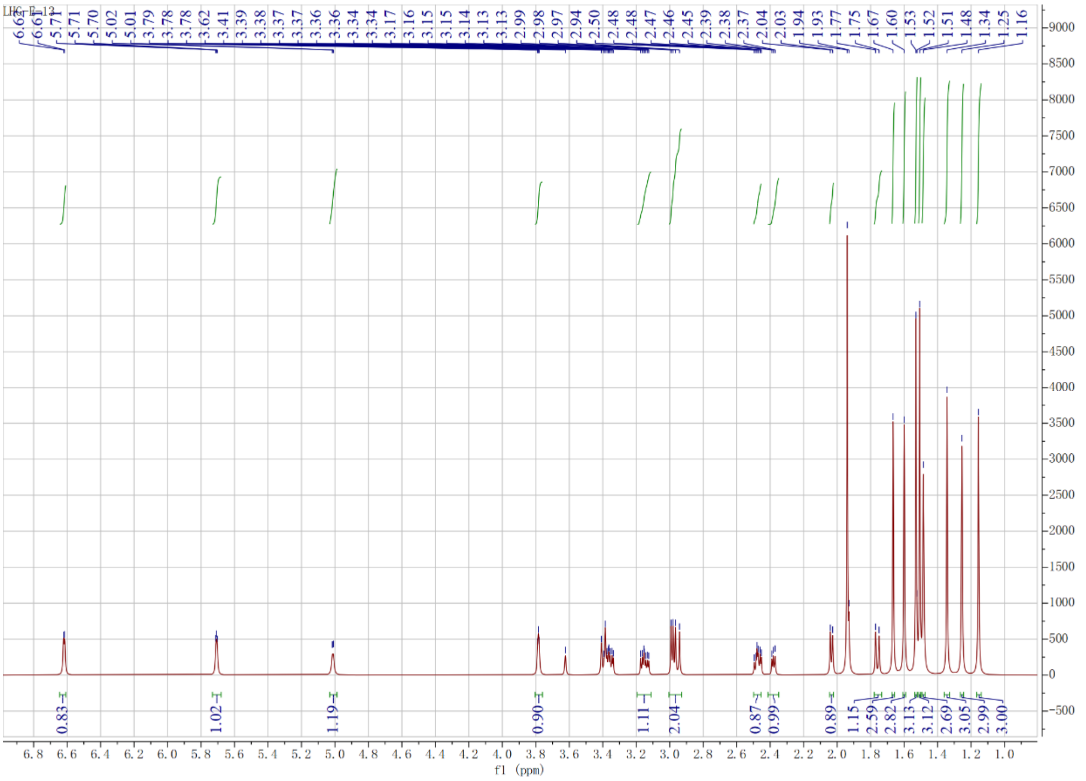

Figure S1 ^{1}H-NMR (600 MHz, pyridine-*d*5) spectrum of 23,24-dihydrocucurbitacin E

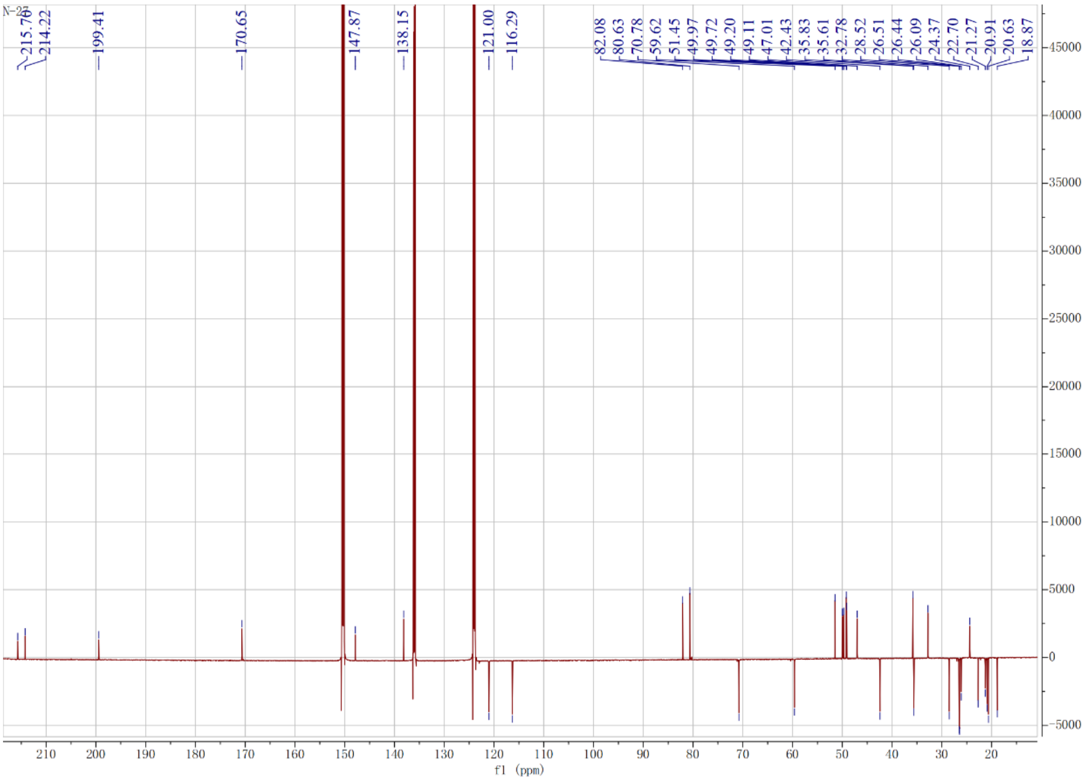

Figure S2 ^{13}C-APT (150 MHz, pyridine-d5) spectrum of 23,24-dihydrocucurbitacin E

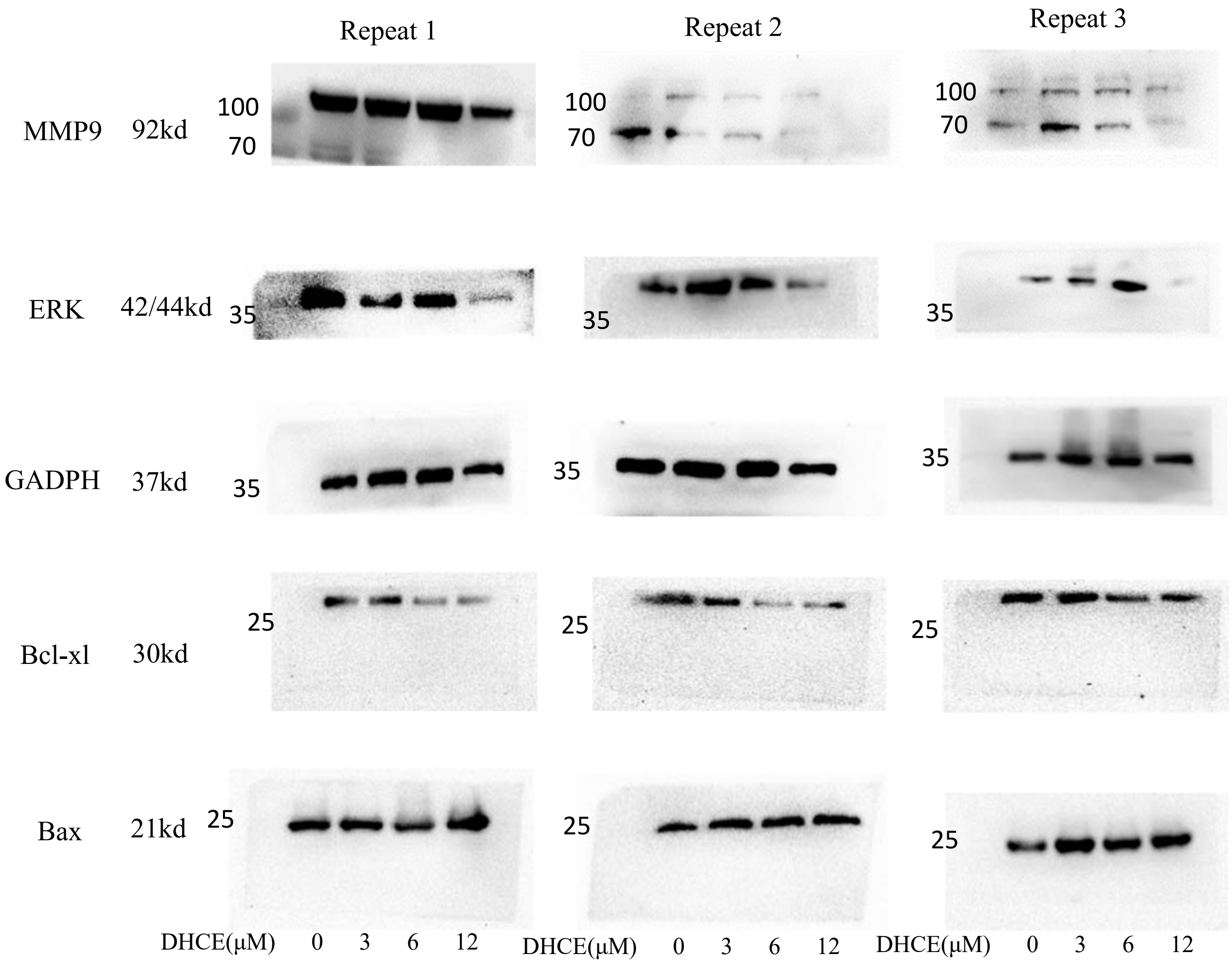

Figure S3 Original western blots images of MMP9, ERK, Bcl-xl and Bax in MGC803 cells (n=3).

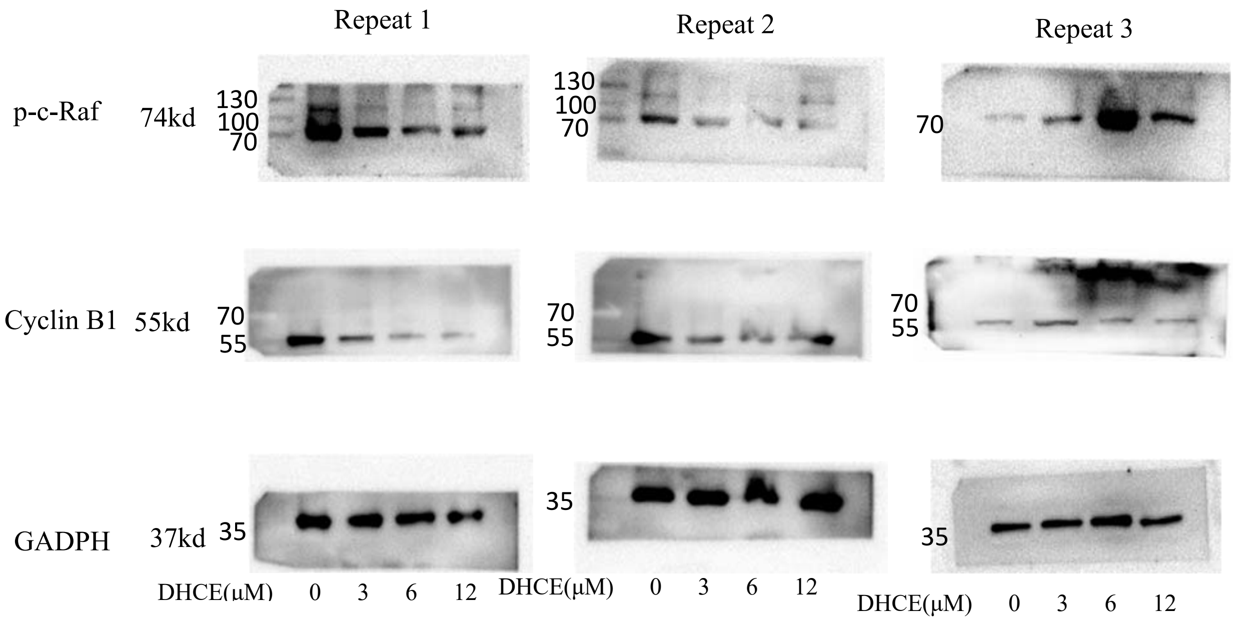

Figure S4 Original western blots images of p-c-Raf and Cyclin B1 in MGC803 cells (n=3).

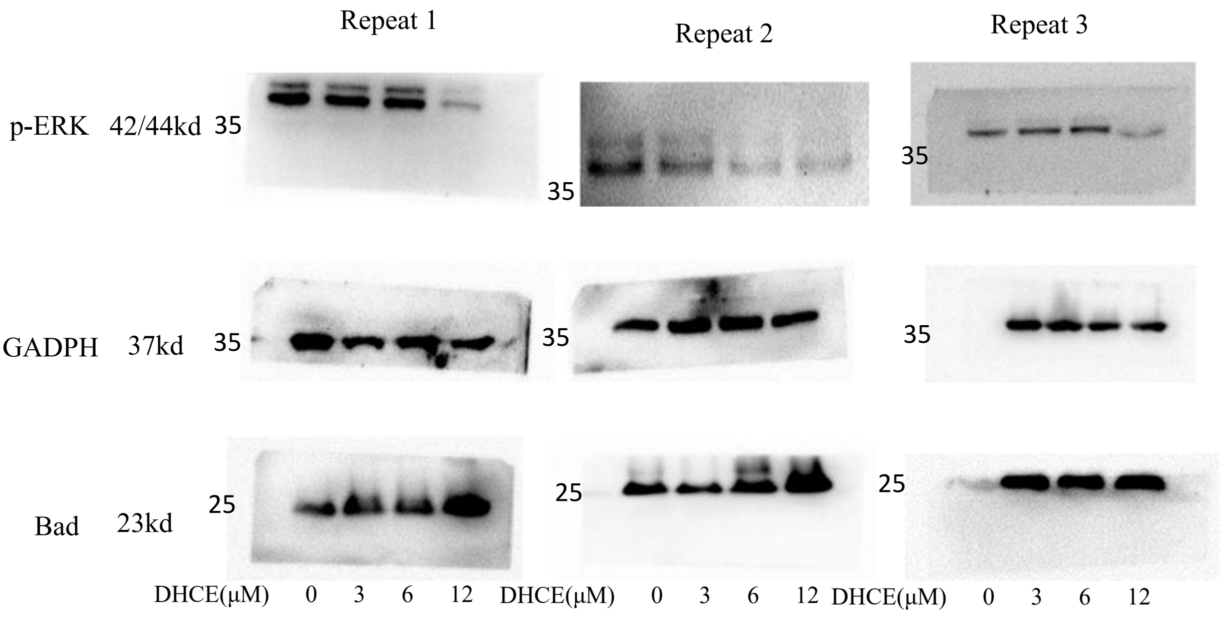

Figure S5 Original western blots images of p-ERK and Bad in MGC803 cells (n=3).

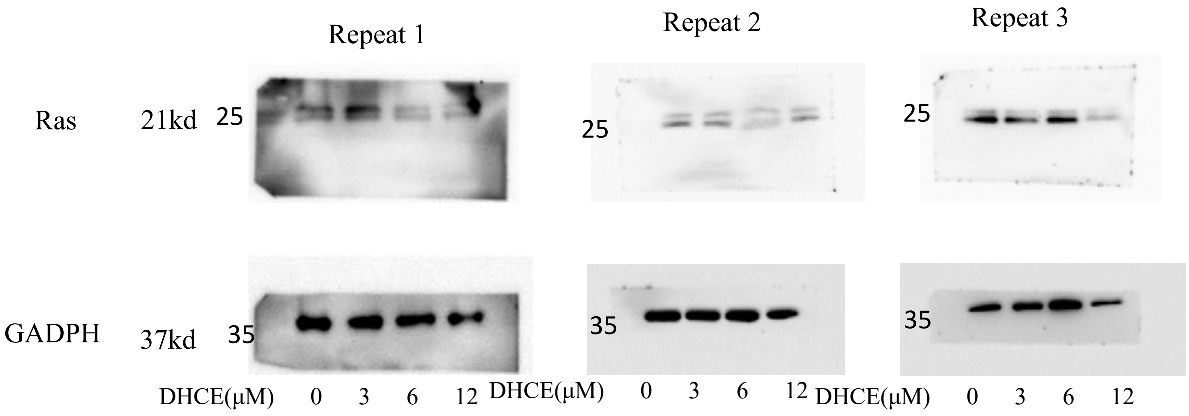

Figure S6 Original western blots images of Ras in MGC803 cells (n=3).

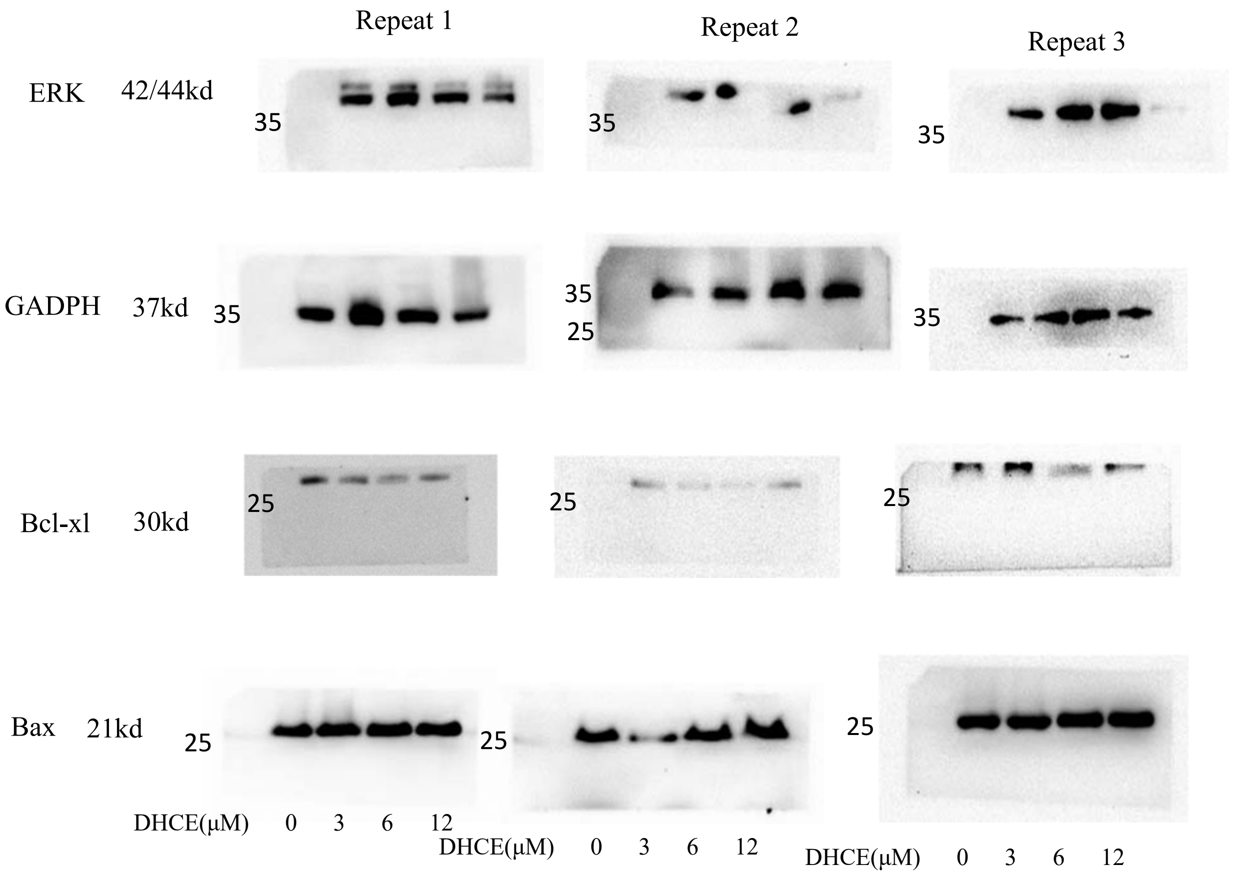

Figure S7 Original western blots images of ERK, Bcl-xl and Bax in SGC7901 cells (n=3).

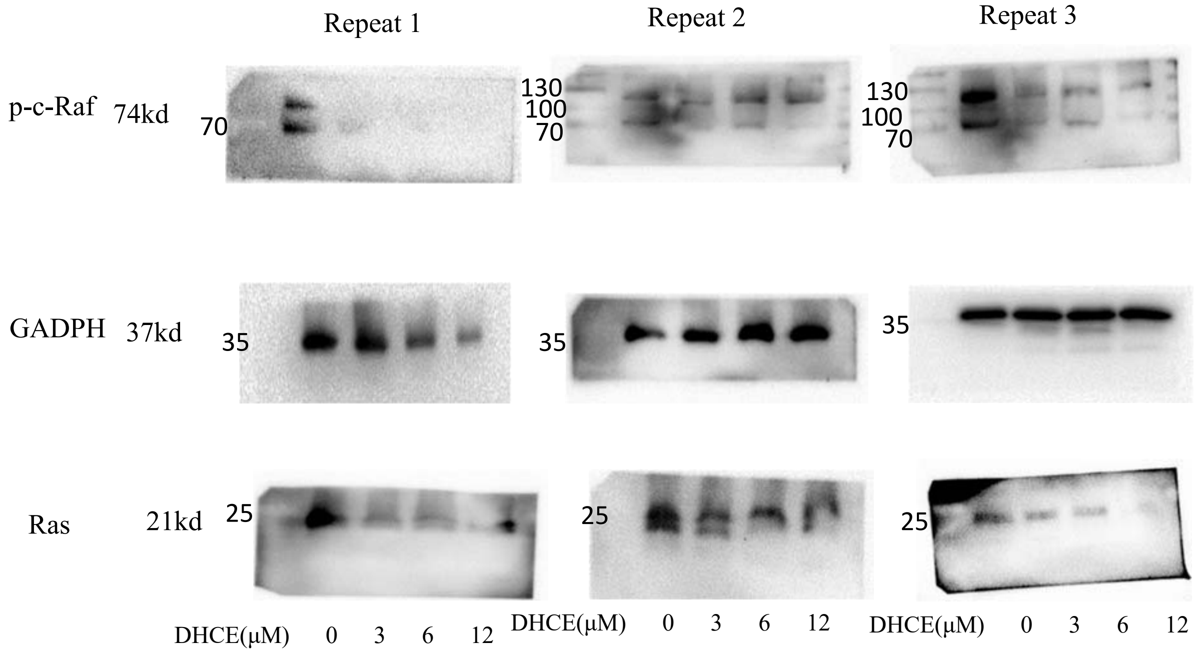

Figure S8 Original western blots images of p-c-Raf and Ras in SGC7901 cells (n=3).

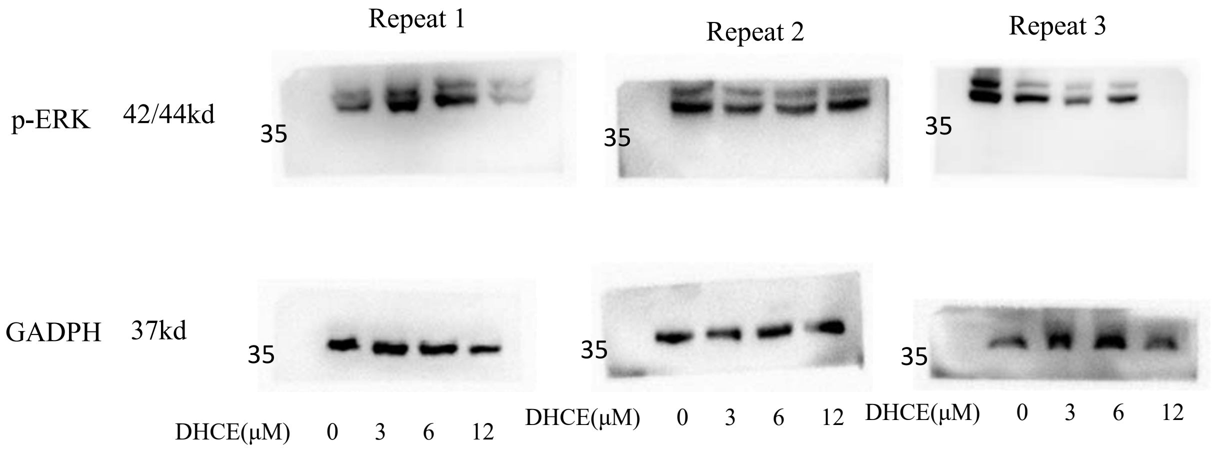

Figure S9 Original western blots images of p-ERK in SGC7901 cells (n=3).

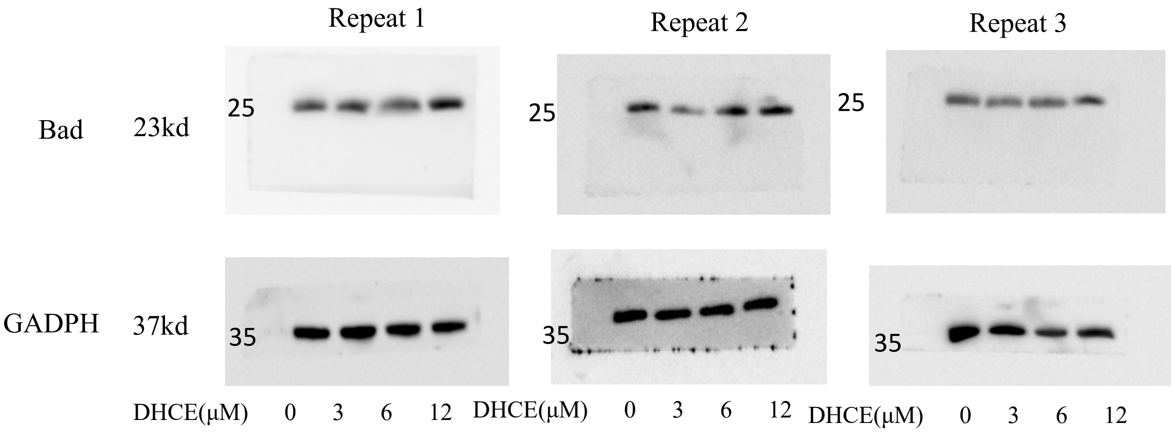

Figure S10 Original western blots images of Bad in SGC7901 cells (n=3).

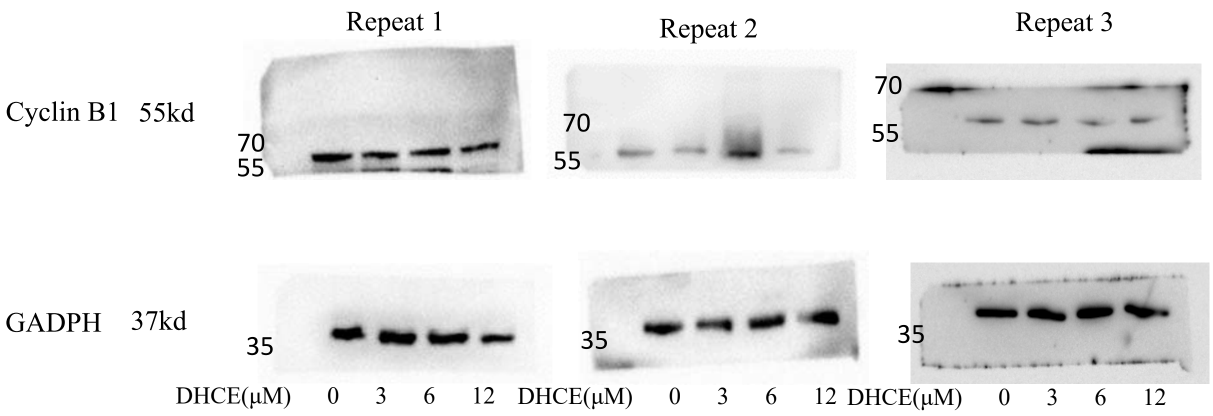

Figure S11 Original western blots images of Cyclin B1 in SGC7901 cells (n=3).

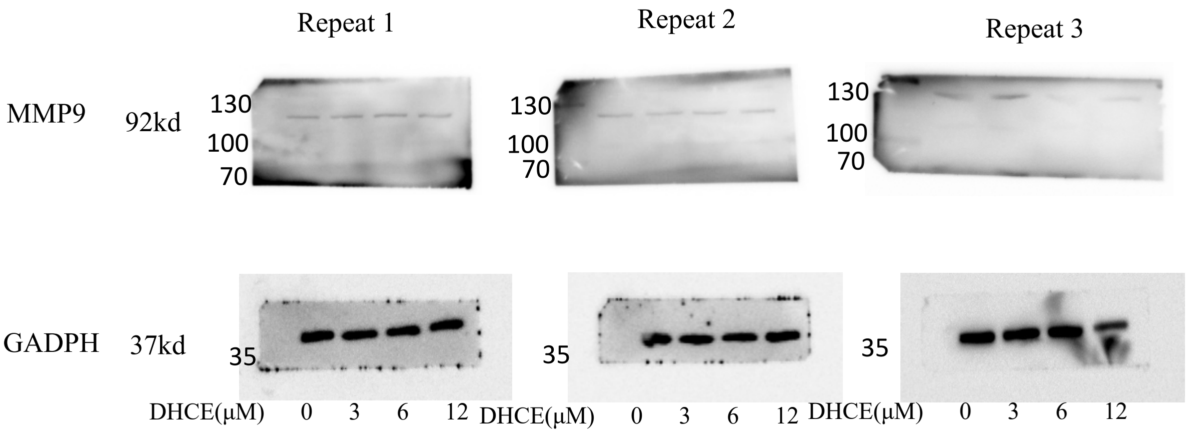

Figure S12 Original western blots images of MMP9 in SGC7901 cells (n=3).
